# Quasiepitaxial Aluminum Film Nanostructure Optimization for Superconducting Quantum Electronic Devices

**DOI:** 10.3390/nano13132002

**Published:** 2023-07-04

**Authors:** Mikhail Tarasov, Andrey Lomov, Artem Chekushkin, Mikhail Fominsky, Denis Zakharov, Andrey Tatarintsev, Sergey Kraevsky, Anton Shadrin

**Affiliations:** 1 V. Kotelnikov Institute of Radio Engineering and Electronics, Moscow 125009, Russia; chekushkin@hitech.cplire.ru (A.C.); demiurge@hitech.cplire.ru (M.F.); 2 Valiev Institute of Physics and Technology, Moscow 117218, Russiadeniszakharovm@mail.ru (D.Z.); tatarintsevandrew@gmail.com (A.T.); 3 V. Orekhovich Institute of Biomedical Chemistry, Moscow 119435, Russia; 4 Moscow Institute of Physics and Technology, Dolgoprudny 141701, Russia; shadrinant@mail.ru

**Keywords:** nanoelectronics, Josephson junctions, nanodevices, crystal structure, epitaxial films, surface roughness, tunnel junctions

## Abstract

In this paper, we develop fabrication technology and study aluminum films intended for superconducting quantum nanoelectronics using AFM, SEM, XRD, HRXRR. Two-temperature-step quasiepitaxial growth of Al on (111) Si substrate provides a preferentially (111)-oriented Al polycrystalline film and reduces outgrowth bumps, peak-to-peak roughness from 70 to 10 nm, and texture coefficient from 3.5 to 1.7, while increasing hardness from 5.4 to 16 GPa. Future progress in superconducting current density, stray capacitance, relaxation time, and noise requires a reduction in structural defect density and surface imperfections, which can be achieved by improving film quality using such quasiepitaxial growth techniques.

## 1. Introduction

The development of superconducting amplifiers, detectors, mixers, and Josephson qubits is limited by different structural defects in metal films, amorphous insulators, and tunnel barriers. In such devices, quantum coherence is required over large distances. Two-level defects in the tunnel barrier can significantly limit the performance of quantum devices. Reducing defect density is required for future progress in relaxation time and noise, which can be achieved by improving film quality using epitaxial growth. Dielectric losses and randomly charged two-level systems (TLSs) are the main origins of energy relaxation and decoherence of Josephson qubits. Another limiting factor for Josephson junctions is the relatively low critical current density combined with a relatively high specific capacitance. According to [1], the effective area of tunneling for Al/AlO_x_/Al junctions is about 0.13 of the geometric area, and the remaining area brings excess stray capacitance. Improving the surface of all layers toward an atomically smooth surface can improve these parameters by nearly an order of magnitude. We previously studied the morphology, roughness, and conductivity of thermally evaporated aluminum films [2] in relation to thickness and contamination. We also studied the crystal structure, surface roughness, and grain structure of magnetron-sputtered aluminum films intended for the fabrication of NIS and SIS tunnel junctions with AlOx or AlN barriers for junctions with Nb, NbN, and Al superconducting electrodes. The morphology and properties of magnetron-sputtered Nb, NbN, and Al superconducting electrodes intended for the fabrication of advanced NIS and SIS tunnel junctions with AlOx or AlN barriers were also investigated [3].

The experimental results are presented in [3], along with roughness and resistivity measurements for magnetron sputtering films at room temperature. Usually, Al films are magnetron-sputtered on Si (100) substrates covered with an insulating layer of Al_2_O_3_. An example of the average layer-by-layer increase in surface roughness, in this case, is presented in Table 1.

The final roughness of the film surface is dependent on the deposition method and can vary in the range of 5–10 nm with a grain size of 100–400 nm when thermally evaporated from a hot boat and by using electron beam evaporation or magnetron sputtering. It is also dependent on the type and crystal orientation of the substrate, as well as on the temperature of the substrate and the deposition rate. Usually, defects in tunnel junctions are associated with defects in metal films and barrier structures, and it is natural to improve the surfaces and nanostructures of basic metal films, which, in most cases, is aluminum. Our main task was to reduce the surface roughness to an atomically smooth surface and obtain an atomically perfect crystal structure. This can be achieved with epitaxial films.

## 2. Fabrication

For smooth epitaxial growth of heterostructures, it is crucial to find a proper substrate material and orientation for single-crystal substrates. At first glance (see Table 2), there is no suitable substrate for the epitaxial growth of Al films.

Actually, the lattice mismatch of 25.3% between Si (111) a = 0.5407 and Al (111) a = 0.404 is accommodated at the interface by alignment of every three Si atoms to four Al atoms. Annealing at 400 °C leads to a reduction in defects in the Al film. 

The heteroepitaxial films of aluminum on sapphire in [4], deposited on a (0001) surface in Bravais indices (equivalent to (111) in Miller indices), provide a (111)-oriented Al film, of which the crystal structure coincides with the sublattice of oxygen. There are two symmetrically equivalent orientations, and this leads to twin defects that prevent the fabrication of atomically smooth films. Molecular beam epitaxy of Al on Si (111) in [5], GaAs(001) in [6], and Al_2_O_3_ in [7] is usually fabricated at a substrate temperature of about 750 °C with an RMS surface roughness down to 1 nm. In fact, peak-to-peak roughness is very important for the fabrication of tunnel heterostructures, which determines the density of the main defects and their strength. Our choice is a (111) silicon substrate and a two-stage deposition process similar to the process of [8] for e-beam evaporation of atomically smooth Ag/Si (111) single-crystal heterostructures. From the beginning, we chose magnetron sputtering as an industrial process, contrary to thermal deposition, which was developed for laboratory applications convenient for shadow evaporation. 

**Figure 1:**
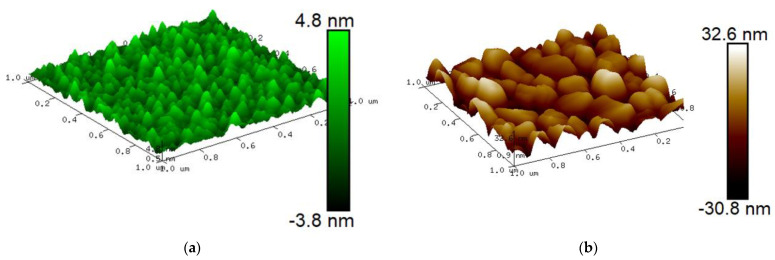

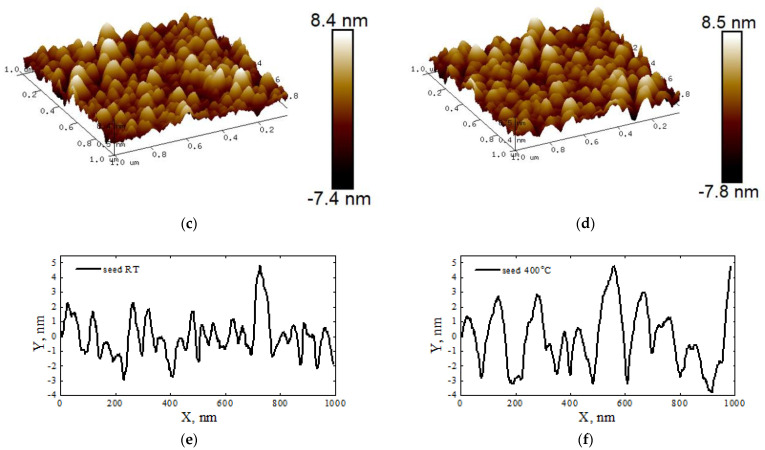
AFM images of 20 nm thick seed layers sputtered on substrate at substrate temperatures of 20 °C (**a**), 200 °C (**b**), 400 °C (**c**), 500 °C (**d**), and cross-sections for 20 °C (**e**) and 400 °C (**f**).

Films 150 nm thick were deposited on a silicon substrate 300 µm thick using a magnetron sputtering system (Kurt J. Lesker Ltd., Dresden, Germany) in the DC discharge mode and a power of 500 W. An aluminum target with a purity of 99.995% was used. Al films were deposited on the substrate both in the one-stage and two-stage modes. In the one-stage mode, the film growth process (ordinary film deposited at room-temperature, or conventional film CF) takes place in an argon atmosphere (pressure 4 × 10^−3^ mbar) at a speed of 1.09 nm/s for 138 s. In the two-stage mode on the substrate at 400 °C there was a preliminary formation of a seed layer at a rate of 0.2 nm/s with a thickness of about 20 nm. After the substrate with seed layer is cooled down to room temperature, in the second stage, the additional layer of the Al film was deposited at a speed of 1.09 nm/s. During the growth of seed films, the temperature of the silicon substrate was maintained at 19 °C by means of automatic water cooling from the substrate stage chiller. After slow cooling over 10 h to 19 °C, the rest of the film was sputtered to a final thickness of 160 nm. We used an atomic force microscope (AFM) and scanning electron microscope (SEM) to examine the seed layer deposited at four temperatures: 20 °C, 200 °C, 400 °C, and 500 °C (see Figure 1).

## 3. Film Nanostructure

**Figure 2:**
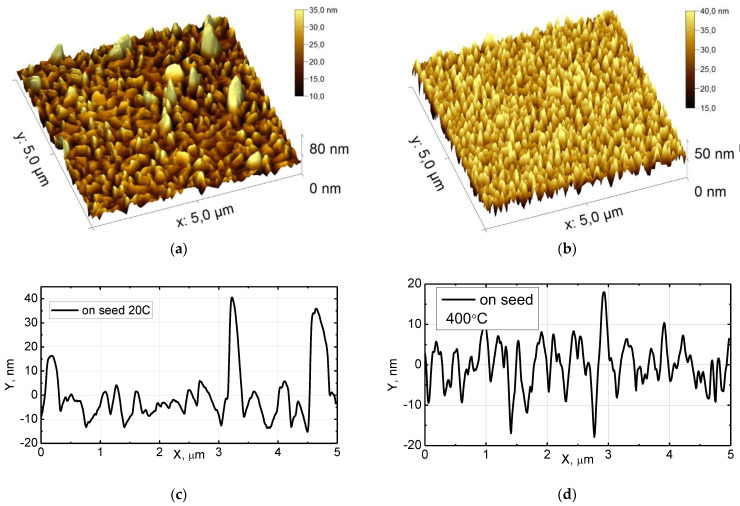
AFM view of 160 nm thick Al films sputtered onto room-temperature substrate (**a**) and above the 400 °C seed layer (**b**). Cross-section of seed layers at room temperature (**c**) and 400 °C (**d**) with p/p roughness 50 nm and 35 nm, respectively.

**Figure 3:**
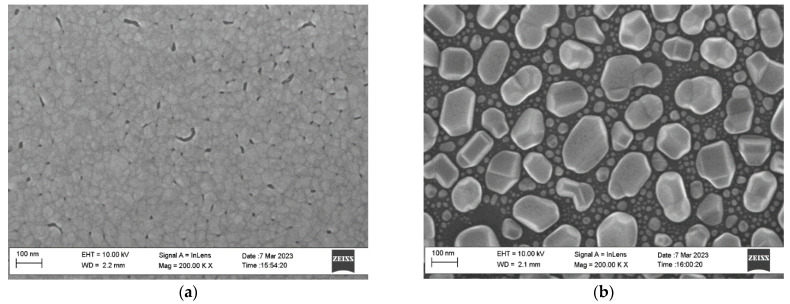

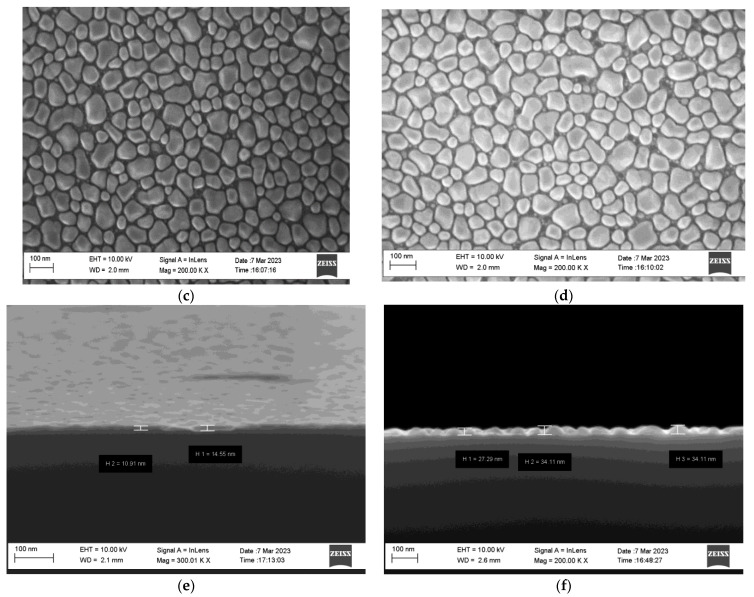
SEM images of 20 nm thick seed layers sputtered on substrates at substrate temperatures of 20 °C (**a**), 200 °C (**b**), 400 °C (**c**), 500 °C (**d**). Corresponding cross-sections (**e**) for 20 °C, (**f**) for 500 °C.

To inspect the fabricated seed layers and final growth on the films, we used a scanning probe microscope (SPM) (also known as an atomic force microscope (AFM)). AFM measurements were performed using a Dimension Icon microscope (Bruker) equipped with commercial RTESPA-300 cantilevers, in QNM mode, in air. The tip of the cantilever is made of Si, with a nominal radius of 8 nm, a resonant frequency of 300 kHz, and a pre-calibrated (Bruker) nominal stiffness of 40 N/m. The scan rate was 1 Hz, and the peak force setpoint was ~3 nN. The AFM images of the samples are shown in Figure 1 for seed layers and Figure 2 for 160 nm thick films with and without hot seed. The average morphological parameters of “cold–hot” seed layers are presented in Table 3. At 20 °C, the seed film is nearly continuously interspersed with voids, and at 200 °C, the film is discontinuous with separated islands of different sizes. At 400 °C and 500 °C, the films are continuously flat with voids, pinholes, and grain boundary grooves, but the islands are relatively uniform with regular shapes (Figure 3). Film type, grain width, and grain height data are presented in Table 4. The outgrowth bumps and peak to peak roughness on the room-temperature substrate are 1.5–2 times smaller compared to the 400 °C seed case.

**Figure 4:**
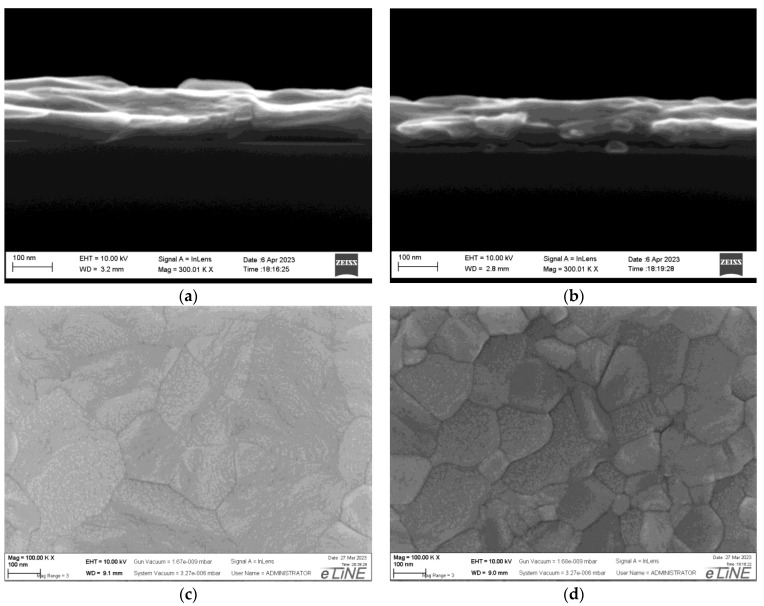
SEM cross-section images of 160 nm thick Al/Si (111) heterostructures sputtered completely on room-temperature substrate (**a**) and above seed layer deposited at T = 400 °C (**b**). Plane view of film deposited at room-temperature (**c**) and above hot seed (**d**).

At the next stage we measured thick films fabricated by sputtering of additional 140 nm aluminum after cooling the seed layer to room temperature. A cross-section of the 160 nm thick samples measured using SEM is presented in Figure 4. The final 160 nm thick Al films were also studied using SEM and X-ray diffraction techniques.

**Figure 5:**
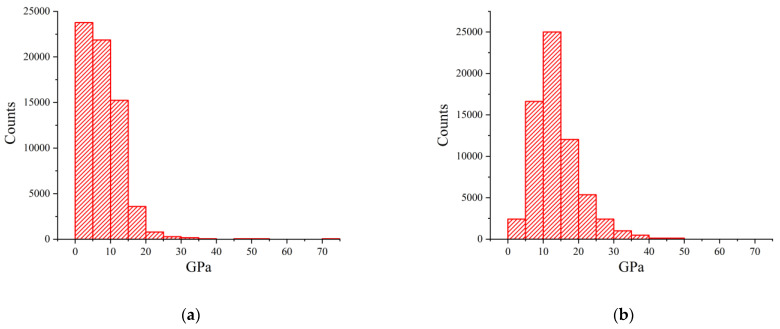
Hardness histogram of the cold (**a**) and hot (**b**) seed films.

Moreover, we also studied the hardness of the films, first using AFM analysis (see Figure 5) and then using a diamond nanoindenter, as shown in Table 5.

It can be concluded that the hardness of films on hot seeds is about three times harder compared to films on room-temperature seed. The hardness of hot seed films exceeds metallurgic bulk aluminum obtained with zone melting. The textbook value of Young’s module for aluminum is 70 GPa; thus, our films above hot seeds are even harder. Excess hardness of multicrystal films can be related to multiple facets of small grains.

**Figure 6:**
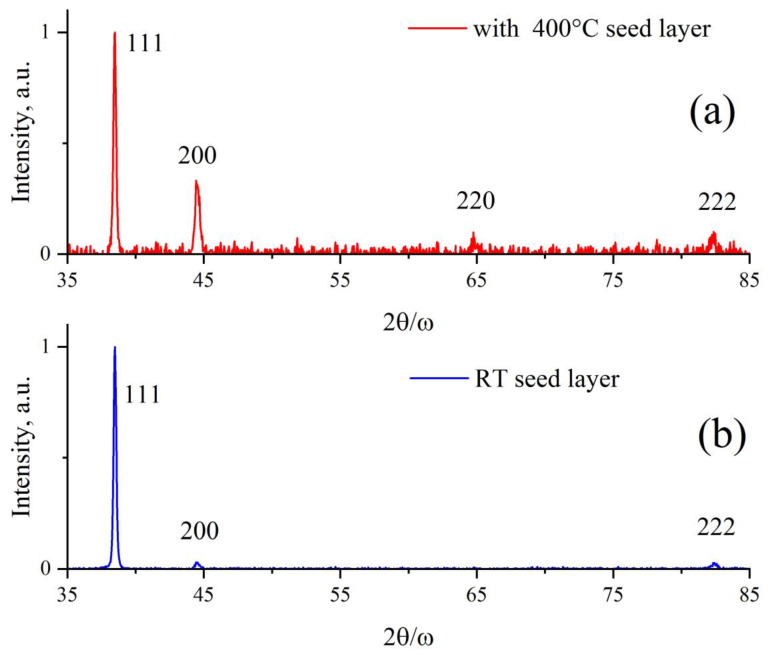

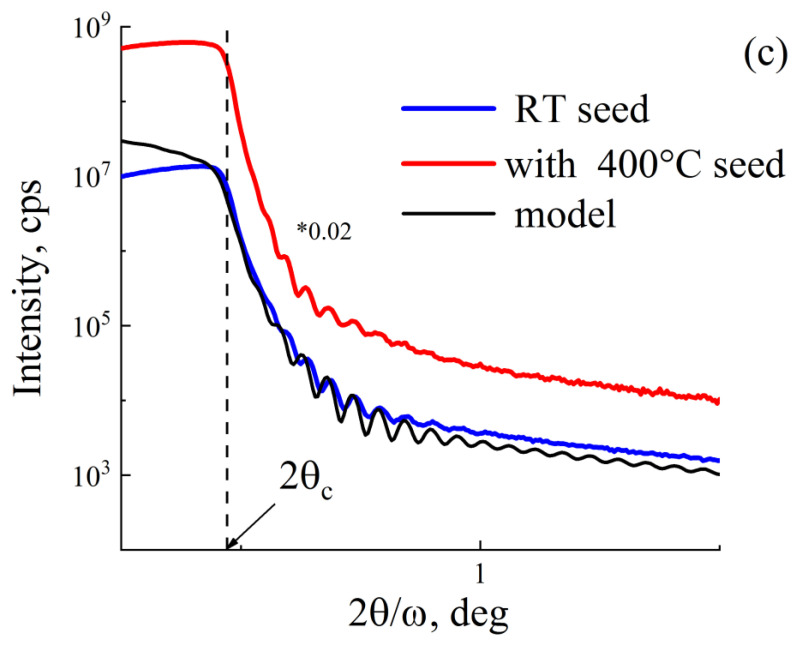
X-ray diffraction (XRD) (**a**,**b**) and high-resolution X-ray reflection HRXRR (**c**) study of room-temperature and “hot” seed films.

More detailed data on the crystal structures were obtained by using XRD and HRXRR, as shown in Figure 6.

The diffraction peaks correspond to a facet-centered lattice with constant a = 0.405 nm. Film on room-temperature seed is more textured, and the texture coefficient P(hkl) for perfect polycrystalline is 1; for conventional Al film on a room-temperature substrate, it is 3.5; and for hot seed film, it is 1.7 (see Table 6). Film above hot seed contains more grain boundaries, it is closer to polycrystalline film with chaotic orientation of crystallites. Room-temperature film is more regular oriented, with less facets on the surface. More faceted (textured) crystal with preferential orientation of crystallites that finally tends to single-crystal, is contrary to random-oriented case with texture coefficient P(hkl) = 1 for perfect polycrystalline film.

An analysis of the maxima half-width shows that it does not correspond to the model of a classical powder with a chaotic arrangement of equal crystallites. The peaks from (111) are 1.5 times narrower than the (200) reflection type. We also collected all the above data from AFM and SEM and compared morphology of seeds and films in Table 7. Variations in film parameters arise from differences in fabrication conditions in two different laboratories with different equipment, from sample to sample in different series, and measurements with different equipment and operators. The important feature is that roughness of cold seed layers is at average twice as low compared to hot seeds. Contrary to that, roughness and grain size of thick films on hot seed is twice as low when compared to thick film on cold seed.

## 4. Discussion

Interest in nanometer-sized films is primarily associated with the possibility of using their electrical and optical properties in microelectronic devices. It turns out that the transition from the creation of three-dimensional elements to film structures can lead to unpredictable changes in the properties of the material used. The main reason for such changes is associated, firstly, with the microstructure of the film and, secondly, with the increasing effect of surface imperfections with a decrease in its thickness. Other factors affecting film properties are the type of substrate used and the method and techniques of film formation. The relationship between the properties of the film and the technology of its deposition on the substrate can be obtained through studies of the real structure. 

Figure 6a,b show the X-ray diffraction patterns from films grown on a cold substrate and on a seed layer deposited at 400 °C (“hot” seed layer). The angular positions of the observed diffraction peaks, marked with crystallographic indices, coincide with the card data (PDXL #01-080-5308) for the polycrystalline Al sample. An analysis of the angular positions of the reflection peaks shows that, in both cases, practically unstrained crystalline films are obtained. However, the ratio of the peak intensities indicates the presence of a pronounced texture (111) in the film (b) in comparison with the texture of the film on the hot seed layer (a). The texture coefficients of the main 111 reflections are P111 = 3.5 and 1.7 for the room-temperature and hot seed films, respectively. It should be noted that the aluminum film grown on the hot seed layer exhibits a larger number of peaks in the diffraction pattern, which reflects the effect of the seed layer on the microstructure of the Al film. A joint analysis of the X-ray diffraction data presented in Figure 6 and Table 6 shows that a polycrystalline microstructure leads to a decrease in the surface roughness of the film.

The HRXRR curves of X-ray reflectometry, which make it possible to determine the thickness of the films and characterize the distortions and roughness of their surface, are shown in Figure 6c. For better visualization, the specular reflection curve from the HBF film sample in Figure 6c is shifted along the y-axis. It can be seen that the critical angles and the period of Kissing oscillations [9] coincide with each other and correspond to the numerical calculations for dense aluminum films of 160 nm thickness. However, the reflection intensity both in the TER region and at large angles differ markedly. Thus, the reflection coefficient of the room-temperature sample in the total external reflection (TER) region is lower than that of the sample with the hot seed film. At the same time, the “tails” of the specular reflection curve of the hot seed sample fall off much faster. In addition, the oscillations on the curve from the hot seed film sample decay faster. This is a manifestation of two different factors on the reflectance roughness at the outer surface and inner boundary of the film with the substrate (for comparison, see the SEM images in Figure 3 and Figure 4). To restore the parameters of the morphology and surface roughness of the grown films, additional studies are required (for example, see [10]).

In our previous study of Al films and tunnel junctions [3], we studied room-temperature deposited Al/AlOx/Al SIS tunnel junctions and SINIS detectors operating at temperatures down to 100 mK. We mentioned that the practical parameters for many Josephson junction devices are much worse compared to theoretical estimations for perfect interfaces with atomically smooth surfaces. For example, for Nb SQUIDs with an AlOx barrier, the characteristic voltage for the nonhysteretic IV curve is below 0.2 mV, contrary to the 2 mV expected in theory. For room-temperature deposited Al tunnel junctions with a specific resistance of 1 kΩ/µm^2^, the capacitance is about 50–70 fF/µm^2^ and the critical current is 0.2 µA/µm^2^, which corresponds to a hysteretic parameter of β_c_ = 32. Thus, for the nonhysteretic operation of Josephson junctions, it should be shunted by a resistance below 33 Ω. Such shunting simultaneously suppresses the voltage response by the same factor. For a flat capacitor C = 50 fF/µm^2^, the barrier thickness should be 0.4 nm. However, a critical current of 0.2 µA/µm^2^ corresponds to a barrier thickness of 1.8 nm, and for such thicknesses, the capacitance should be 15 fF/µm^2^. These estimations clearly show that both the effective area and the thickness of the barrier are different from the oxide thickness estimation and geometric area.

The characteristic value for capacitive hysteresis is the McCumber parameter β_c_ [11], which is a dimensionless admittance ratio, β_c_ = ω_c_C/G = ω_c_RC, or in parameters of junction β_c_ = τ_c_/τ_L_ = RC × R (2eI_c_/ħ). Here, we take asymptotic resistance instead of dynamic because V_c_ = I_c_R_n_. The capacitance of a flat sandwich-type capacitor C = ε_0_εS/d with ε_0_ = 8.85 × 10^−12^ F/m, d = 1 nm, S = 1 µm^2^, ε_1_ = 11.5, or ε_2_ = 9.4 for crystal Al_2_O_3_ is C_1_ = 101 fF and C_2_ = 83 fF. In some practical cases, this oxide can be amorphous and loose with a reduced dielectric constant and lower capacitance. However, with a surface roughness as in Figure 1, the area of the capacitor can be twice as much compared to the geometric area. For practical cases with an amorphous barrier ε_0_ = 4, the capacitance can be estimated in the range of 50–80 fF/µm^2^.

The tunneling current and the resistance of tunnel junctions are determined by the mechanism of electron tunneling. The transmission of the tunneling barrier [12] can be presented as the ratio of forward and transmitted wave function amplitudes:

$$\frac{F}{A}=4\sqrt{\frac{E_{0}}{E}\;}exp\left(-\frac{\sqrt{2mE_{0}}}{h}w\right)$$

where *E*_0_ is the barrier height, *E* is the energy of an electron, *m* is the mass of an electron, and *w* is the barrier width. Conductivity is proportional to the square of the amplitudes. For *E*_0_ = 1 eV and *w*_1_ = 1 nm, the ratio of conductivities is (*F*/*A*)^2^ = 4 × 10^−4^, and for w_2_ = 2 nm, it is four orders lower than (*F*/*A*)^2^ = 10^−8^, the same as the ratio of resistances R_2nm_/R_1nm_ = 4 × 10^4^. However, for these two very different conductivities, the capacitance C_w1_ is only twice as large compared to C_w2_. 

According to [13], variations in the barrier thickness produce the “hot spot” regions that concentrate the tunnel current. For example, for an oxide layer thickness of 4 nm measured by TEM, the tunneling thickness extracted from conductance is 1 nm. The effective area can be up to five orders of magnitude smaller than the geometric area of the junction. The oxide thickness measured by XRD and TEM increases from 4.2 nm (T_ox_ = 10 s) up to 6.4 nm (T_ox_ = 50 s), whereas the tunneling thickness varies from 1 to 1.9 nm. As estimated by [14], the ratio of capacitance thickness and tunneling thickness can be up to 10^6^. If conductivity is determined by a number of hot spots, then we can assume that such spots on oxide tips can carry a unit of electrical conductance G_0_ = 2e^2^/h = 7.748 × 10^−5^ S or R_0_ = h/2e^2^ = 12.9 kΩ. If the tunnel junction contains 10–100 such contacts, the parallel resistance can be in the range of 1.3–0.13 kΩ. Additionally, the rest of the junction area impacts only capacitance. If we take an average Al_2_O_3_ grain size of 100 nm with a 111 orientation (vertex at the top), this results in 100 tips on an area of 1 × 1 µm^2^ and, in the best case, 100 parallel junctions with a total resistance of 130 Ω. Longer oxidation leads to a reduction in the number of conducting junctions, according to the distribution of height. Similar assumptions in [15] in terms of superconducting quantum point contacts (SQPCs) provide an estimation for the conductivity of such a single channel as σ = 2e^2^D/h with a channel transparency of D = 0.7. The number of SQPS can be determined by the surface roughness of the barrier as well as the additional channels induced by electric stress. As was mentioned in [16], for a satisfactory explanation of the IV curves of high-transparency junctions, the presence of electron channels through the barrier with a transmission probability > 0.5 is required.

Contrary to hot spot and SQPS models, the pure theory of ideal flat tunnel junction provides the exact values without fitting parameters. The relations for tunnel junction resistance without fitting parameters [17] takes into account the image potential, which reduces the corners of the potential barrier. The zero bias resistivity is RS = $\frac{t}{\sqrt{2mE_{0}}}\exp\left(\frac{4\pi t}{h}\sqrt{2mE_{0}}\right)$, or in practical units RS = $\frac{t}{3\cdot 10^{10}\sqrt{E_{0}}}\exp\left(t\sqrt{E_{0}}\right)$ (Ω·cm^2^), where t (angstroms) is the barrier thickness and *E*_0_ (volts) is the barrier height. For t = 1 nm and *E*_0_ = 4 V, this gives RS = 10^−6^ Ω·cm^2^ or 100 Ω·µm^2^, the same as for *E*_0_ = 2 V and t = 1.5 nm. This estimation is surprisingly close to previous estimations for quantum conductance. 

The important role of film morphology and improvements in junction quality by means of epitaxial growth was demonstrated in [18]. The NbN/AlN/NbN trilayers were continuously epitaxially grown on single-crystal MgO (100) substrates by using reactive dc-magnetron sputtering. The AlN barriers were deposited at a low power of 0.3 W/cm^2^ and a deposition rate of about 0.05 nm/s. The obtained values for barrier thicknesses of 2 nm are a quality factor of R_sg_/R_n_ = 60 and a current of density J_c_ = 0.22 µA/µm^2^, and for barrier thicknesses of 1 nm, the quality factor was reduced to R_sg_/R_n_ = 10 and the current density was increased to J_c_ = 250 µA/µm^2^. Earlier investigations of AlN barriers in [19] clarify the average barrier height, which is 2.3 eV for low-transmission barriers that correspond to a thick AlN layer with hexagonal structures and 0.88 eV for low-transmission thin (<1 nm) barriers with cubic crystal structures in close vicinity to the cubic Nb underlayer. These facts confirm the need to study the crystal structures of tunnel barriers. 

The importance of studying interface roughness is illustrated in [20], where the effective barrier thickness is 1 nm for a barrier of 1.5 nm thickness and 15% roughness. The effective thickness is nearly two-and-a-half standard deviations below the mean thickness. Moderate amounts of roughness cause the conductance to resemble that of much thinner and taller barriers.

For the perfect Al tunnel junction area of S = 1 µm^2^ with 1 nm barrier thickness, the critical current should be 36 µA, the resistance 5.6 Ω, V_c_ = 196 µV, C = 24 fF, β_c_ = 0.014, the time constant RC = 0.13 ps, and the corresponding cutoff frequency 1 THz. In practice, such parameters were never achieved; with a reduction in the barrier oxidation time, the quality factor decreases, and microshorts dominate. Improvements in the surface roughness and crystal structure of Al films should substantially increase the current density, characteristic voltage, and frequency and reduce relative specific capacitance and hysteresis, as well as reducing 1/f noise. The example of single-crystalline Al films in [5] proves that with proper substrate treatment it is possible grow heteroepitaxial structure across the entire 4” wafer without grain boundaries. 

## 5. Conclusions

We developed a fabrication technology and improved the roughness of aluminum films intended for superconducting quantum nanoelectronics. Reducing the structural defect density and surface distortion (imperfections or unevenness) is required to achieve the theoretically predicted characteristics of superconducting devices. The two-temperature-step quasiepitaxial growth of Al on (111) Si substrate provides a preferentially (111)-oriented Al polycrystalline film and reduces outgrowth hills, peak-to-peak roughness from 50 to 20 nm, and the texture coefficient from 3.5 to 1.7, while increasing hardness from 5.4 to 16 GPa. Future progress in cryogenic devices, both increasing superconducting current density and decreasing stray capacitance, relaxation time, and white noise, can be achieved by improving film quality using such quasiepitaxial growth techniques. 

## Figures and Tables

**Table 1:** Surface roughness for layers of room-temperature deposited Al film.

Layer	Roughness (rms)	Treatment
Si (100) substrate	0.5 nm	Conventional
Al_2_O_3_ 100 nm film	3.2 nm	Magnetron sputtering
Al 60 nm film	7.6 nm	Magnetron sputtering

**Table 2:** Crystal structures and lattice constants for Al and dielectric materials.

Material	Syngony	Lattice Constant, nm Angles, Deg.	T Melting, °C	Density, g/cm^3^
Al	Cubic	a = b = c = 0.404	660	2.70
Al_2_O_3_	Hexagonal Trigonal	a = b = 0.4759, c = 1.299 α = β = 90, γ = 120, a = b = c = 0.518, α = β = γ = 55.27	2050	3.99
SiO_2_	Trigonal r-form z-form Hexagonal	a = b = c = 0.31 α = β = γ = 85.2° α = β = γ = 94.8° a = b = 0.413, c = 5.404, α = β = 90°, γ = 120°	1713	2.65
AlN	Hexagonal	a = b = 0.311, c = 0.498, α = β = 90°, γ = 120	2200	3.26
MgO	Cubic	a = b = c = 0.420 α = β = γ = 90	2825	3.58
Si	Cubic	a = b = c = 0.5407 α = β = γ = 90	1414	2.33

**Table 3:** AFM-based morphology parameters of magnetron-sputtered 20 nm thick Al seed films on Si (111) substrate.

Growth T, °C	Quality	Grain Width nm	Roughness, Peak-to-Peak, nm	Roughness rms, nm
20	Continuous	30	3	0.6
200	Insular	160	25	6.6
400	Insular	60	10	1.6
500	Insular	60	5	0.8

**Table 4:** Parameters of 20 nm seed layers deposited at different temperatures, extracted from SEM.

Temperature, °C	20	200	400	500
Film type	Continuous	Insular	Insular	Insular
Grain width, nm	20–50	100–200	40–80	50–100
Grain height, nm	11–14	42–50	20–27	27–34

**Table 5:** The hardness of Al/Si (111) films sputtered on room temperature and 400 °C seeds, as well as without seed.

Seed	Hardness, GPa	Young’s Module, GPa
400 °C seed	16	95
20 °C seed	5	35

**Table 6:** Texture and roughness of 20 °C- and 400 °C-seeded Al/Si (111) films from XRD studies.

Sample Al/Si (111)	Texture (111)	Roughness rms, nm	Roughness, Peak-to-Peak, nm
20 °C	3.5	6.3	50
400 °C seed	1.7	6.3	10

**Table 7:** Cumulative data on morphology of thin seed and thick final aluminum films from AFM and SEM measurements.

Seed Temperature		20 °C	400 °C
Seed	Grain size, nm	20–50	60–70
	Rmax, nm	3–6	5–10
	Rms, nm	0.6–1	0.8–1.6
Thick film	Grain size, nm	300–400	70–200
	Rmax, nm	25–40	10–20
	Rms, nm	8–25	6–10

## Data Availability

The details regarding the data supporting reported results can be obtained from M. Tarasov by direct request.
